# Cerebral Amyloid Angiopathy: A Case Report

**DOI:** 10.7759/cureus.84989

**Published:** 2025-05-28

**Authors:** Fatima Alam, Anup Banerjee, Chaminda Jayawarna

**Affiliations:** 1 Acute Medicine, Stepping Hill Hospital, Stockport NHS Foundation Trust, Stockport, GBR

**Keywords:** alzheimer’s dementia, beta- amyloid, brain biopsy, cerebral amyloid angiopathy, intracranial haemorrhage

## Abstract

Cerebral amyloid angiopathy (CAA) is a cerebrovascular condition characterized by the buildup of beta-amyloid protein within the walls of small and medium-sized blood vessels in the brain’s cortex and leptomeninges. Clinically, it can present with a range of neurological symptoms, including recurrent headaches, cognitive disturbances, seizures, and focal deficits. A key feature of CAA is the tendency for lobar brain hemorrhages, though its clinical and radiological profile can overlap with other neurological disorders such as Alzheimer’s disease, demyelinating conditions, vascular syndromes, and neoplasms. CAA pathology is frequently observed in individuals with Alzheimer’s disease, with a subset showing significant vascular involvement. Although histopathological confirmation remains definitive, advanced imaging, particularly MRI, has become central to diagnosis, often identifying features such as cortical microbleeds, superficial siderosis, and non-deep (lobar) hemorrhages. In some presentations, especially inflammatory variants, patients may benefit from immunosuppressive treatment such as corticosteroids, making early diagnosis critical. Recognizing the distinction between CAA-related hemorrhages and other causes of cerebral bleeding is vital for timely and appropriate management. We present a case of an elderly male who had multiple hospital presentations with unexplained self-resolving expressive dysphasia, seizure, and gradually increasing confusion with the possibility of underlying dementia that responded well to high-dose steroids. This case underscores the need to consider inflammatory cerebral amyloid angiopathy (iCAA) as a differential in patients with recurrent, unexplained neurological symptoms with CT evidence of micro- and macro-hemorrhages and a dramatic response to steroids for symptomatic improvement. Improved awareness of atypical presentations and the utility of MRI can support earlier intervention. Further investigation is needed to refine diagnostic tools, identify reliable biomarkers, and explore therapeutic strategies targeting the underlying vascular and inflammatory mechanisms of the disease.

## Introduction

Cerebral amyloid angiopathy (CAA) is a cerebrovascular disorder characterized by the progressive deposition of beta-amyloid protein in the walls of small- and medium-sized cortical and leptomeningeal blood vessels [1]. This pathological process weakens the vascular structure, making affected vessels prone to rupture and leading to recurrent lobar intracerebral hemorrhages. While CAA is commonly associated with aging and Alzheimer’s disease (AD), it can also present as a distinct clinical entity, sometimes manifesting with inflammatory features or rapidly progressive neurological decline [2].

The clinical presentation of CAA is highly variable, ranging from asymptomatic microbleeds detected incidentally to life-threatening intracerebral hemorrhages. Patients may present with recurrent headaches, seizures, cognitive impairment, behavioral changes, or focal neurological deficits [3]. Given the overlap with other neurological conditions such as hypertensive hemorrhage, primary CNS vasculitis, and neoplastic lesions, diagnosis can be challenging [4]. Although histopathological confirmation via brain biopsy remains the gold standard, non-invasive imaging, particularly susceptibility-weighted MRI, has become a crucial tool in recognizing characteristic features such as cerebral microbleeds, cortical superficial siderosis, and lobar hemorrhages [5]. The Boston Criteria, which are widely adopted for classifying CAA as definite, probable, or possible based on clinical, radiological, and pathological findings, are crucial in guiding diagnosis and research.

Timely recognition of CAA is essential, as some variants, particularly those with an inflammatory component, may respond to immunosuppressive therapy [3]. Misdiagnosis or delayed treatment can result in irreversible neurological damage. In this case report, we present a patient with recurrent, unexplained neurological symptoms and radiological findings suggestive of CAA. This case highlights the importance of maintaining a high index of suspicion for CAA in patients with atypical cerebral hemorrhages and underscores the potential for clinical improvement with appropriate early intervention.

## Case presentation

An 80-year-old Caucasian male was brought to the hospital by his wife due to a progressive decline in cognition, confusion, and reduced mobility over six months. His past medical history was unremarkable except for well-controlled hypothyroidism with levothyroxine. He was a lifelong nonsmoker with no history of alcohol misuse. Although he was awaiting assessment by the memory clinic for suspected dementia, his wife reported a concerning recent deterioration following a motor vehicle accident two weeks earlier, in which he drove into a wall. Since the incident, he had become increasingly confused, exhibited word-finding difficulty, and spoke more slowly.

He also complained of new-onset diplopia and difficulty reading. His general practitioner suspected a transient ischemic attack and empirically commenced on aspirin; however, there was no clinical improvement. He denied fever, headache, nausea, behavioral disturbances, or focal limb weakness.

On neurological examination, the patient had diplopia when looking toward the right temporal field, although extraocular movements were intact. He demonstrated features of expressive dysphasia. There were no additional cranial nerve deficits or motor, sensory, or cerebellar abnormalities. The mini-mental state examination (MMSE) score was 22/30. General physical examination was otherwise unremarkable. Laboratory investigations, including erythrocyte sedimentation rate (ESR), C-reactive protein (CRP), full blood count, renal and liver function, glucose, electrolytes, and thyroid profile, were all within normal limits except for mild anemia and borderline hyponatremia (Table 1).

**Table 1:** Laboratory results for the patient INR: international normalized ratio; ESR: erythrocyte sedimentation rate

Parameter	Patient value	Reference value
White Cell Count	5.8 x 10^9/L	3.7 - 11.0 x 10^9/L
Hemoglobin	120 g/L	130 - 180 g/L
Platelet	196 x 10^9/L	150 - 450 x 10^9/L
C-reactive Protein	<4 mg/L	0.0 - 10.0 mg/L
INR	1.1	0.8-1.2
ESR	5 mm/h	1 - 34 mm/h
Serum Sodium	133 mmol/L	133 - 146 mmol/L
Serum Potassium	4.2 mmol/L	3.5 - 5.3 mmol/L
Serum Urea	5.6 mmol/L	2.5 - 7.8 mmol/L
Serum Creatinine	74 umol/L	62 - 115 umol/L

**Figure 1:**
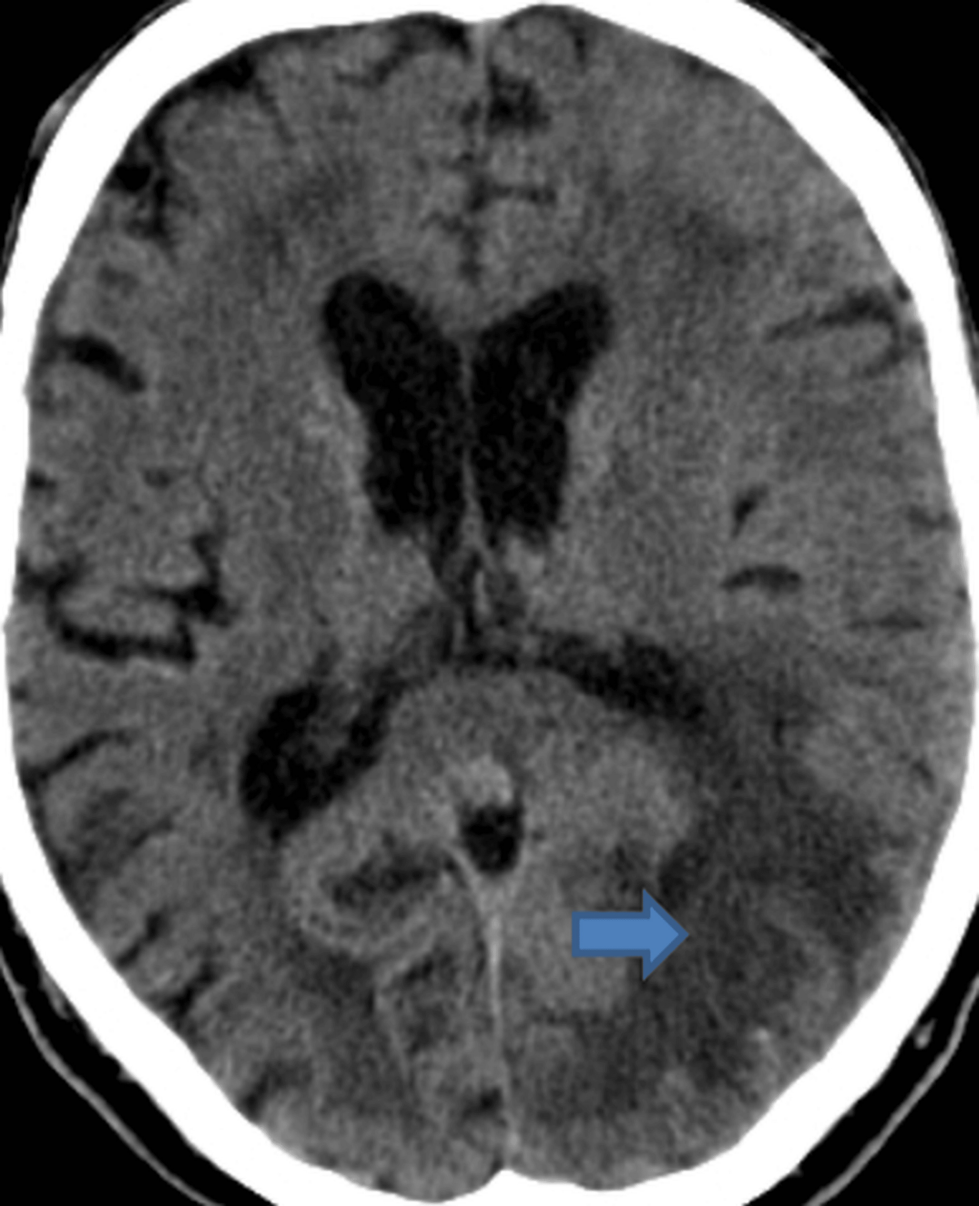
CT head of the patient The arrow is pointing to a region of hypoattenuation in the parieto-occipital region.

A non-contrast CT scan of the head revealed a chronic left middle cerebral artery (MCA) territory infarct, with diffuse swelling and sulcal effacement throughout the left hemisphere, most pronounced in the parieto-occipital region, suggestive of a possible acute-on-chronic infarction, but could not exclude inflammation or infection. Reduction in caliber posterior horn left ventricle, but otherwise normal ventricles and basal cisterns (Figure 1).

However, in the absence of focal neurological deficits, an acute ischemic event was deemed unlikely. The case was discussed with a tertiary neurology team, who raised suspicion for viral encephalitis. Intravenous acyclovir was initiated, and further investigations, including lumbar puncture (LP) and brain MRI, were performed.

LP findings showed significantly elevated cerebrospinal fluid (CSF) protein (3.75 g/L; normal range: 0.15-0.6 g/L) with normal glucose. The CSF cell count of RBC was high due to a traumatic sample (Table 2).

**Table 2:** Laboratory test for the patient PCR: polymerase chain reaction; VZV: varicella-zoster virus; HSV: herpes simplex virus

Parameter	Patient value	Reference value
Appearance	Clear	Clear
CSF Total Protein	3.75 g/L	0.15 - 0.60 g/L
CSF Glucose	3.0 mmol/L	2.2 - 4.4 mmol/L
CSF Lactate	1.9 mmol/L	1.4 - 2.6 mmol/L
HSV1 PCR	Not detected by PCR	Absent
HSV2 PCR	Not detected by PCR	Absent
VZV PCR	Not detected by PCR	Absent
Enterovirus RNA PCR	Not detected by PCR	Absent
Parechovirus RNA PCR	Not detected by PCR	Absent
CSF Micro and Culture	No growth	No growth
CSF RBC Cell Count	298 x10^6/L	0 x10^6/L
CSF WCC Cell Count	<1 x10^6/L	0-5 x10^6/L

**Figure 2:**
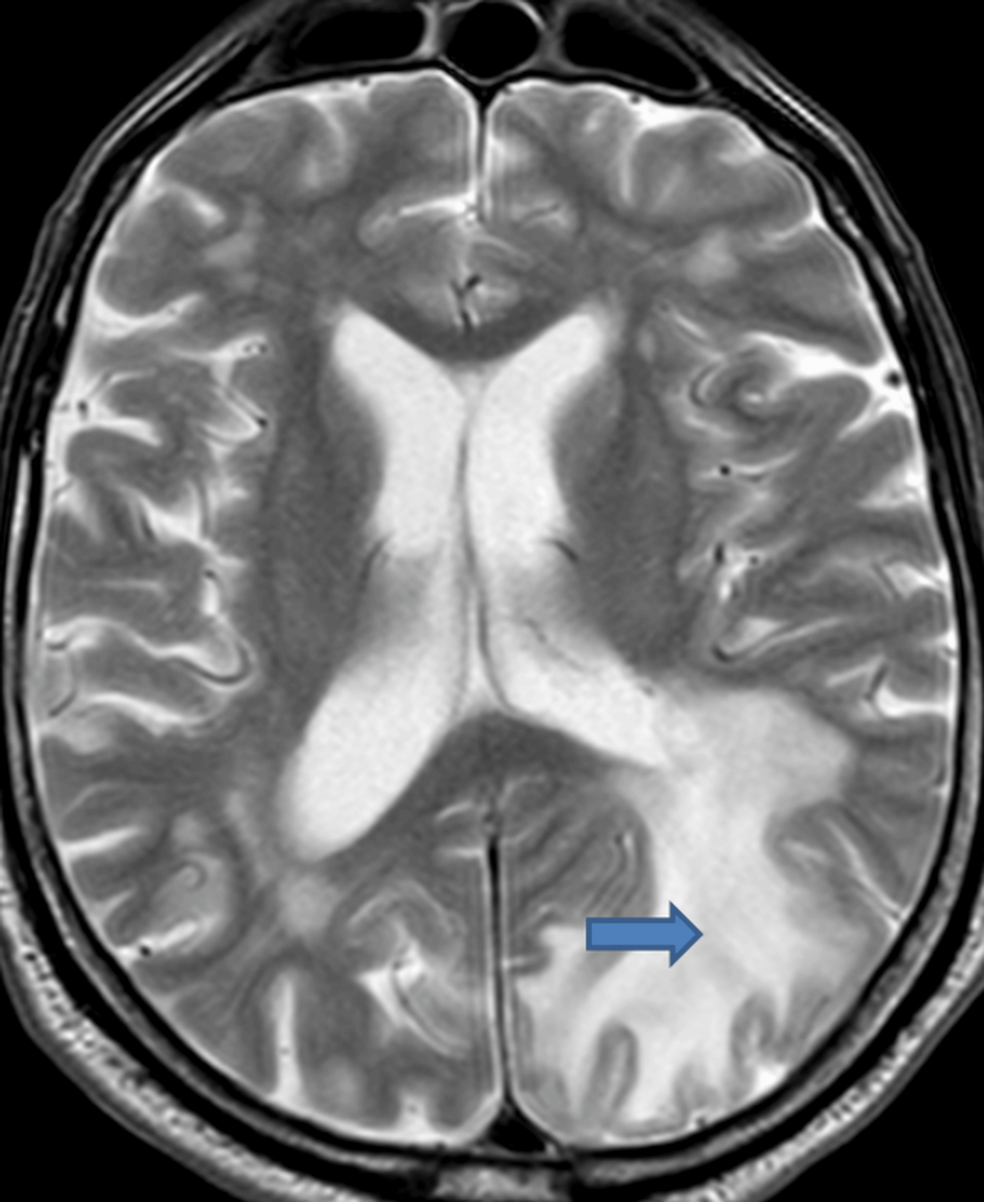
MRI head of the patient The arrow is pointing to an area of hyperintensity in the temporo-parieto-occipital lobe, likely suggestive of edema.

Viral polymerase chain reaction (PCR) was negative, and acyclovir was discontinued. MRI of the brain revealed diffuse signal abnormalities in the left posterior temporal, parietal, and occipital lobes, associated with confluent vasogenic edema and sulcal effacement but no midline shift. Multiple petechial microhemorrhages were also identified within the affected regions (Figure 2).

After liaising with the neurology team, a working diagnosis of inflammatory cerebral amyloid angiopathy (iCAA) was made, and the patient was commenced on oral prednisolone 50 mg daily alongside a proton pump inhibitor and bone protection. Within five days of treatment, the patient showed marked clinical improvement, with resolution of diplopia, improved speech fluency, and reduced confusion. He was discharged after nine days of steroid therapy with a reduced dose of oral prednisolone from 50 mg every three weeks with outpatient neurology follow-up. At that time, his MMSE score was 27/30, which indicates improvement in cognition. 

**Figure 3:**
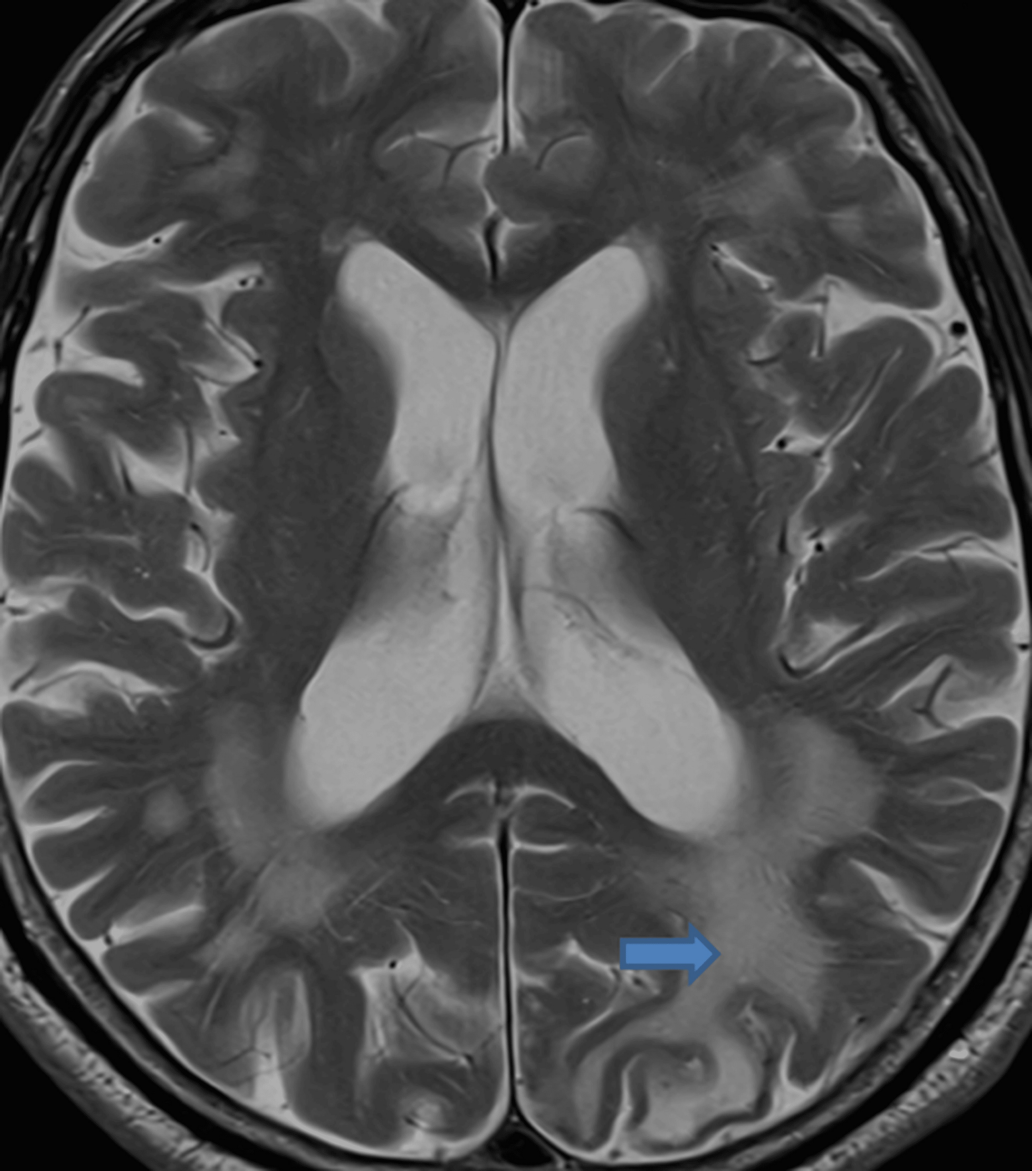
MRI head after six months The arrow is pointing to a marked reduction of hyperintensity in the occipital lobe.

A repeat MRI performed six months later demonstrated significant resolution of the previous encephalopathic changes in the left cerebral hemisphere (Figure 3). However, numerous cortical microhemorrhages persisted, consistent with a diagnosis of CAA with associated inflammatory changes.

## Discussion

CAA is an increasingly recognized cerebrovascular disorder characterized by the accumulation of beta-amyloid protein in the walls of small- and medium-sized cortical and leptomeningeal blood vessels [1]. This pathological deposition compromises vessel integrity, predisposing to recurrent cerebral hemorrhages, most notably in the lobar regions, and is a leading cause of non-traumatic intracerebral hemorrhage in the elderly [3].

Clinically, CAA can present with a wide spectrum of neurological symptoms, including recurrent headaches, transient focal neurological episodes, behavioral changes, seizures, cognitive impairment, and varying degrees of altered consciousness [2,6]. The hallmark of CAA is spontaneous lobar hemorrhage, which often recurs and may be associated with cortical superficial siderosis and cerebral microbleeds. However, due to its variable presentation, CAA can be easily misdiagnosed, as it shares overlapping features with several neurological conditions such as AD [4], progressive multifocal leukoencephalopathy (PML), posterior reversible encephalopathy syndrome (PRES), primary CNS vasculitis, and intracranial neoplasms, including metastases [6].

The pathological presence of CAA has been identified in nearly all patients with AD, with advanced CAA found in approximately 25% of AD brains [7]. This strong association suggests a shared pathogenic mechanism involving beta-amyloid metabolism and deposition, though the precise trigger for vascular vs. parenchymal deposition remains unclear.

Definitive diagnosis is histopathological, requiring brain biopsy or autopsy tissue demonstrating vascular amyloid deposition [2]. However, biopsy is rarely performed due to its invasiveness and associated risk, particularly in elderly patients. In living patients, MRI plays a central role in diagnosis. Susceptibility-weighted imaging (SWI) or gradient-echo T2*-weighted imaging sequences can reveal characteristic findings such as multiple cortical-subcortical microhemorrhages, superficial siderosis, and large lobar hemorrhages [5]. These findings support a diagnosis of “probable” or “possible” CAA based on the Boston Criteria, which have been widely adopted in clinical practice [8].

An important and potentially reversible variant of the disease is iCAA, which includes amyloid-beta-related angiitis (ABRA). This form is characterized by additional perivascular or transmural inflammation, often leading to more rapid neurological decline. Our patient demonstrated classic radiological features suggestive of iCAA, including asymmetric vasogenic edema, sulcal effacement, and associated microhemorrhages, with no evidence of infection or neoplasm. Notably, CSF analysis revealed elevated protein without pleocytosis, further supporting a non-infectious inflammatory process.

Timely recognition of iCAA is critical, as it is one of the few CAA variants that respond well to immunosuppressive therapy. High-dose corticosteroids are the mainstay of treatment, typically leading to rapid clinical and radiological improvement. In our case, the patient exhibited significant improvement in cognition, language function, and visual symptoms within days of initiating steroid therapy. Literature suggests that approximately 75% of patients with iCAA show favorable responses to corticosteroid therapy, often avoiding further neurological deterioration [9].

Prognosis in CAA varies widely and depends on the clinical presentation, age, and extent of hemorrhagic burden. Outcomes tend to be poorer in elderly patients, particularly those presenting with large or recurrent intracerebral hemorrhages [10]. Nevertheless, early identification and management, especially of inflammatory subtypes, can significantly improve functional outcomes and quality of life.

This case highlights the importance of considering CAA in older patients presenting with atypical or unexplained neurological symptoms and radiological findings of lobar hemorrhage or vasogenic edema. It also underscores the role of MRI in distinguishing CAA from its mimics and the therapeutic potential of immunosuppression in inflammatory variants.

Future research should focus on the development of reliable non-invasive biomarkers for early detection and disease monitoring, the refinement of diagnostic criteria for iCAA, and the exploration of alternative immunomodulatory therapies. As the population ages and the incidence of CAA rises, greater awareness and clinical vigilance are essential for improving diagnostic accuracy and patient outcomes.

## Conclusions

This case underscores the diagnostic complexity and clinical significance of CAA, particularly its inflammatory variant. In elderly patients presenting with unexplained neurological symptoms and characteristic imaging findings, such as lobar hemorrhages, vasogenic edema, and cortical microhemorrhages, CAA should be considered as a differential diagnosis. Early identification and prompt initiation of immunosuppressive therapy, especially corticosteroids, can lead to substantial clinical improvement and may prevent long-term neurological deterioration. Clinicians should maintain a high index of suspicion for CAA in patients with overlapping features of neurodegenerative, vascular, and inflammatory disorders. As our understanding of this condition evolves, further research into its pathophysiology, diagnostic tools, and treatment strategies will be crucial to improving outcomes for affected individuals.
